# Lipid lowering in coronary artery disease – not just statins

**DOI:** 10.1016/j.clinme.2026.100594

**Published:** 2026-05-08

**Authors:** Jennie Han, Eva Masmanian, Timothy Wang, Tuan Peng Chua

**Affiliations:** a St George’s Hospital, Blackshaw Road, London SW17 0QT, UK; b Novum Health, The Primary Care Centre, Hawstead Rd, London SE6 4JH, UK; c Royal Surrey County Hospital, Egerton Road, Guildford GU2 7XX, UK

**Keywords:** Coronary artery disease, Lipids, Secondary prevention, Clinical pharmacology, Cardiology

## Abstract

Coronary artery disease has a high degree of morbidity and mortality internationally and, after a cardiovascular event, patients require intensified management of modifiable risk factors, with optimal lipid control being an important cornerstone of secondary prevention. This is both a primary and a secondary care responsibility. Familiarity with indications for and prescription of non-statin medications, including injectables, is vital for patient outcomes. Fewer than 30% of patients reach LDL-c targets with statins alone. This is a public health matter, and non-statin therapies that all clinicians need to be aware of are oral therapies such as ezetimibe and bempedoic acid, and injectable therapies such as inclisiran and PCKS9 inhibitors. Icosapent ethyl is also available in those with high fasting triglycerides with specific LDL-c values. Understanding when to refer to the lipid clinic enables patients to be managed appropriately in the correct setting. Future therapies under development include cholesterol ester transfer protein inhibitors, oral PCSK9 inhibitors, and gene editing therapy targeting ANGPTL3 and PCSK9. Lipid optimisation leads to significant reduction in major adverse cardiac events, and familiarity with all available treatment options – including non-statins and injectables – will facilitate best primary and secondary care.

## Graphical Abstract

**Figure:**
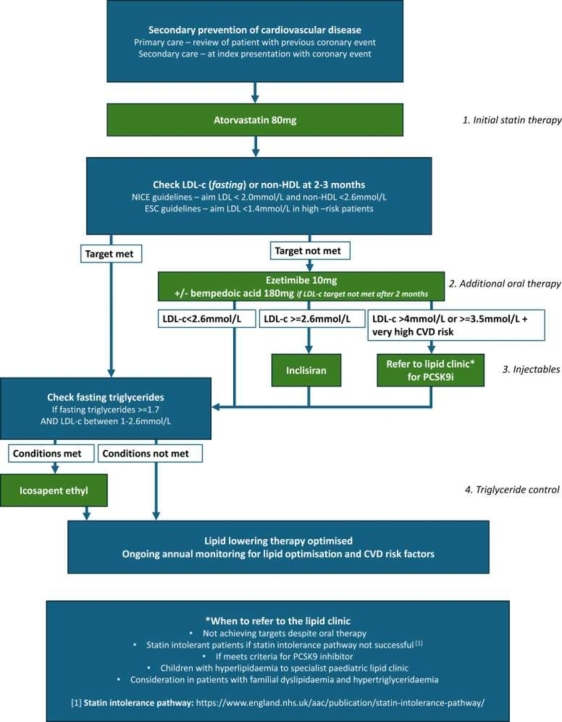
Summary diagram of prescribing and optimising lipid-lowering therapy in secondary prevention. Four stages include initial statin therapy, followed by additional oral therapy, then injectables, and then triglyceride control. PCSK9 inhibitors have to be initiated in the lipid clinic in the UK. Guidance for when to refer to the lipid clinic is included. Abbreviations: CVD, cardiovascular disease; ESC, European Society of Cardiology; NICE, National Institute of Health and Care Excellence; PCSK9i, PCSK9 inhibitor.

## Background: lipid levels and coronary artery risk

Coronary artery disease has a high degree of morbidity and mortality internationally. Within the UK, there is a large socioeconomic burden estimated at £29 billion annually, based on costs of intervention, medications, inpatient and outpatient care, cardiac rehabilitation and indirect costs.^1^

After a cardiovascular event, patients require intensified management of modifiable risk factors, with optimal lipid control being an important cornerstone of secondary prevention. Trials have shown that lowering low-density lipoprotein cholesterol (LDL-c) correlates with a reduction in major adverse cardiac event (MACE) rates; every 1 mmol/L reduction in LDL-c reduces the risk of MACE by 21%.^2^ This has been demonstrated through landmark randomised controlled trials for statin and proprotein convertase subtilisin/kexin type 9-inhibitor (PCSK9i) therapy.^3^, ^4^, ^5^, ^6^ No trial of lipid-lowering medications has yet shown harm from overreduction of cholesterol.^7^, ^8^

Lipid optimisation is both a primary and a secondary care responsibility. The European Society of Cardiology (ESC) 2025 guidelines recommend that, for patients admitted with an acute coronary syndrome who are already taking lipid-lowering treatment, therapy should be intensified during the same hospital admission to further reduce LDL-c to <1.4 mmol/L (Table 1). Familiarity with non-statin medications including injectables is vital for patient outcomes; the Department of Health, UK has reported that by treating 300,000 patients annually with inclisiran, an injectable lipid-lowering agent, 55,000 heart attacks and strokes can be prevented over 10 years.^9^

**Table 1:** Lipid targets in secondary prevention for the European Society of Cardiology (ESC) and National Institute for Health and Care Excellence (NICE). Note that the ESC recommends tighter LDL-c targets, whereas NICE guidelines are based on cost-effectiveness, and their economic model suggests that an LDL-c target of 1.8 mmol/L was not cost-effective, hence a higher target. NICE also recommends non-HDL-c as this is easier to deliver, as it can be done in non-fasting lipid samples.

	ESC guidelines	NICE guidelines
Established cardiovascular disease	LDL-c <1.4 mmol/L or >50% reduction	LDL-c ≤2.0 mmol/L or >50% reduction Non-HDL-c ≤2.6 mmol/L or >40% reduction
Recurrent vascular event within 2 years	LDL-c <1.0 mmol/L	

Surrogate endpoints in the form of LDL-c lowering exist for trials of all approved lipid-lowering therapies discussed in this review (Table 2).^10^ Hard outcomes (MACE reduction) exist for all approved lipid-lowering therapies except inclisiran, where completed MACE outcome data are awaited.

**Table 2:** Extent of lipid lowering with available therapies.^10^

Agent	Dose	Approximate reduction in LDL-c
Fluvastatin	20 mg	21%
	40 mg	27%
	80 mg	33%
Pravastatin	10 mg	20%
	20 mg	24%
	40 mg	29%
Simvastatin	10 mg	27%
	20 mg	32%
	40 mg	37%
	80 mg (not recommended due to muscle toxicity)	42%
Atorvastatin	10 mg	37%
	20 mg	43%
	40 mg	49%
	80 mg	55%
Rosuvastatin	5 mg	38%
	10 mg	43%
	20 mg	48%
	40 mg	53%
Ezetimibe 10 mg		24%
Ezetimibe 10 mg + bempedoic acid 180 mg		38%
Inclisiran		50%
Alirocumab (PCSK9i)		50%
Evolocumab (PCSK9i)		50%

## Oral therapy

Statins are the first-line treatment for lipid-lowering therapy, reducing *de novo* synthesis via inhibition of HMG-CoA reductase. Although muscle symptoms attributed to statins are often explained by nocebo effects or alternative causes,^11^, ^12^ statins can cause myalgia, deranged liver function and rhabdomyolysis, so may not always be tolerated. In many cases, rechallenging with statins at a lower dose and appropriate patient counselling of side effects may help with patient compliance. However, in many patients LDL-c targets are not reached with statin therapy alone.^13^, ^14^ In fact, fewer than 30% of these patients achieve LDL-c targets.^14^, ^15^

If lipid targets are not met or if statins are not tolerated, ezetimibe and bempedoic acid are secondary prevention options indicated for LDL-c >2 mmol/L or non-HDL-c >=2.6 mmol/L.^4^, ^16^, ^17^ The main mechanism of action of ezetimibe is reducing small intestine absorption of cholesterol, at a dose of 10 mg once a day. Bempedoic acid inhibits adenosine triphosphate citrate lyase, which then reduces cholesterol synthesis in the liver. Bempedoic acid 180 mg once a day is only approved by NICE as combination therapy with ezetimibe. Trials have demonstrated an additional LDL-c reduction of 24% with ezetimibe and 28% with ezetimibe and bempedoic acid combination therapy,^18^, ^19^ with associated decrease in MACE.^4^, ^16^, ^17^

## Injectables

After statins, injectable lipid-lowering agents are the most potent methods of lowering LDL-c.^5^, ^6^, ^20^, ^21^, ^22^ However, these are underutilised due to lack of awareness of availability and indications.^14^ The initiation of both inclisiran and PCSK9i is dependent on a patient’s LDL-c levels, measured as part of a fasting lipid screen. Inclisiran can be started at a lower LDL-c threshold, but PCSK9i have a high LDL-c threshold for initiation, due to the cost of these medications and resultant cost-effectiveness calculations.

PCSK9i blocks the PCSK9 enzyme, increasing cell surface LDL receptor concentration. There are two PCSK9i monoclonal antibodies available, evolocumab and alirocumab, which are initiated in specialist clinics and self-administered subcutaneously either every 2 or 4 weeks. These are indicated for secondary prevention if LDL-c >4 mmol/L or >3.5 mmol/L with recurrent events. In familial hypercholesterolaemia (FH), the indication is LDL-c >5.0 mmol/L for primary prevention or 3.5 mmol/L for secondary prevention. These are associated with an additional LDL-c reduction of 50%, and the FOURIER and ODYSSEY OUTCOMES trials demonstrated decreases in MACE in secondary prevention.^5^, ^6^ Side effects include injection site reactions, hypersensitivity, nausea, arthralgia and, rarely, angioedema.

Inclisiran is a small interfering RNA (siRNA) that targets PCSK9 mRNA in the liver, decreasing its production. It is licensed for patients with a history of cardiovascular events and an LDL-c >=2.6 mmol/L despite maximally tolerated lipid-lowering therapies. After two initial doses at 1 month and 3 months, maintenance is with 6-monthly 284 mg subcutaneous injections. Side effects are uncommon, usually related to injection site reaction. NHS England is funding inclisiran centrally from a national NHS budget to improve access via primary care: inclisiran is available in GP practices at a nominal cost of £45 per injection and will be reimbursed at £60.^23^ Inclisiran trials have demonstrated an LDL-c reduction of 50% at 30 days, which is maintained over 18 months.^20^, ^21^, ^22^ However, there have been no hard MACE outcomes reported with inclisiran, and there has been some criticism from the Royal College of General Practitioners and British Medical Association on the initial NICE approval.^24^ ORION 4 and ORION 5 are due to report this in 2026.

## Practical considerations

After a cardiac event, patients should be initiated on a high-dose statin such as atorvastatin 80 mg once a day. This should be reviewed usually within 2–3 months with a lipid profile to see if therapy needs to be intensified.

If injectable therapies are to be considered, a fasting lipid profile should be performed to obtain LDL-c levels, as these are required for all injectable treatments.

Patients with statin-associated myopathy, confirmed by a creatine kinase (CK) level of over five times the upper limit of the normal range, as well as those with intolerable symptoms, should undergo a ʻdechallenge’ and ʻrechallenge’ according to the statin intolerance pathway.^25^ In the event of a failed rechallenge, the next step should be a trial of ezetimibe +/− bempedoic acid, or inclisiran if eligibility criteria are met. Note that if renal function is affected or if CK is over 50 times the upper limit of normal, rhabdomyolysis should be considered and specialist advice urgently sought.

NICE does not support the use of co-enzyme Q10 to treat statin-related myalgia, although a recent small systematic review of one meta-analysis and four randomised control trials showed some benefit in the 800 patients analysed.^26^

The effect of lipid-lowering therapy in a developing fetus is unknown and currently it is recommended that all such therapies are stopped, ideally 3 months prior to conception and throughout the breastfeeding period.

## Special cases

Fibrates and bile acid sequestrants are available for patients with FH not achieving targets with statins or ezetimibe. Evinacumab is a monoclonal antibody that blocks the angiopoietin-like 3 protein (ANGPTL3) and is licensed by NICE in homozygous FH at a cost of £6,433/vial for intravenous infusion given every 4 weeks.^27^

Hypertriglyceridaemia has been implicated as an independent predictor of risk.^28^, ^29^ Fibrates are the most effective agents for reducing triglycerides, but NICE has suggested that they should not be routinely used for reducing cardiovascular risk except in FH. Omega 3 fatty acids are also not recommended by NICE for risk reduction, and primary care clinicians are advised not to use them due to a possible association with atrial fibrillation. Fibrates and omega 3 fatty acids should be considered in severe hypertriglyceridaemia when there is a risk of acute pancreatitis. The main effect of icosapent ethyl is reduction in triglycerides rather than LDL-c. It is indicated in secondary prevention in those only with *fasting* triglycerides ≥1.7 mmol/L *and* taking statins with an LDL-c between 1.04 and 2.6 mmol/L.^30^, ^31^ Triglycerides should not be based on a random non-fasted lipid sample.

## When to refer to the lipid clinic

A lipid clinic referral can be made if the patient is not achieving targets despite standard therapy (statins, ezetimibe, and bempedoic acid). Statin-intolerant patients should also be referred if the statin intolerance pathway has not been successful.^26^ Currently, the PCSK9 inhibitors, evolocumab and alirocumab, must be initiated in secondary care and so, if eligible, these patients will also require a lipid clinic referral. It is important to ensure that they fulfil the NICE criteria for this therapy prior to referral.^32^, ^33^

Patients who may have a familial dyslipidaemia should be considered for referral to a specialist lipid clinic. The presence of tendon xanthomata, eyelid xanthelasma and corneal arcus is suggestive of FH. The Simon Broome criteria or the Dutch Lipid Clinic Network (DLCN) criteria are used to aid clinical diagnosis of FH in primary care. A clinical diagnosis of FH can be used in those with Simon Broome criteria for ‘possible’ or ‘definite’ FH (Table 3), or have a DLCN score greater than 5 (Table 4).^34^ On the basis of NICE guidelines from 2008, patients with FH were seen in a specialist clinic.^35^ However, FH is a very common condition and the workload for lipid clinics has escalated in recent times such that many clinics can no longer manage this workload. As a consequence, some local guidelines encourage primary care doctors to make the initial diagnosis using LDL-c and family history, initiate treatment with high-intensity statin and ezetimibe and only refer if this is not successful (ie reduction of LDL-c <50% from pre-treatment baseline level). Referral to a cascade service for gene testing and family screening is also desirable, accessed either via direct referral from primary care or via lipid clinics according to local pathways. Children with hyperlipidaemia should be referred to a specialist paediatric lipid clinic.

**Table 3:** Simon Broome criteria for ‘definite’ and ‘possible’ familial hypercholesterolaemia.^34^

	Lipid criteria	AND one of:
‘Definite’ FH in child/young person	Total cholesterol >6.7 mmol/L or LDL-c >4.0 mmol/L	• Tendon xanthomata in the person with suspected FH, a first- or a second-degree relative • DNA-based evidence of a LDL receptor mutation, familial defective apo B-100, or a PCSK9 mutation
‘Definite’ FH in adult	Total cholesterol >7.5 mmol/L or LDL-c >4.9 mmol/L	
‘Possible’ FH in child/young person	Total cholesterol >6.7 mmol/L or LDL-c >4.0 mmol/L	• Myocardial infarction in first-degree relative aged <60 years or second-degree relative aged <50 years • First-degree or second-degree relative with total cholesterol >7.5 mmol/L (if adult) or >6.7 mmol/L (if aged <16 years)
‘Possible’ FH in adult	Total cholesterol >7.5 mmol/L or LDL-c >4.9 mmol/L

**Table 4:** Dutch Lipid Clinic Network criteria to diagnose familial hypercholesterolaemia (FH). Score >8 for definite FH, 6–8 for probable FH, 3–5 for possible FH, and <3 for unlikely FH.^34^

	Score
**Family history**	
First-degree relative with known premature coronary/vascular disease (men aged <55 years, women aged <60 years) *or* First-degree relative with known LDL-c above 95th percentile for age and sex	1
First-degree relative with tendon xanthomata and/or corneal arcus *or* Children aged <18 years with LDL-c above 95th percentile for age and sex	2
**Clinical history**	
Premature coronary artery disease (men aged <55 years, women aged <60 years)	2
Premature cerebral or peripheral vascular disease (men aged <55 years, women aged <60 years)	1
**Physical examination**	
Tendon xanthomata	6
Corneal arcus before age 45 years	4
**Investigations**	
LDL-c ≥8.5 mmol/L	8
LDL-c 6.5–8.4 mmol/L	5
LDL-c 5.0–6.4 mmol/L	3
LDL-c 4.0–4.9 mmol/L	1
**DNA analysis**	
Functional mutation in LDL receptor, apolipoprotein B or PCSK9 gene	8

Extreme hyperlipidaemia should also be a consideration for referral. In particular, hypertriglyceridaemia should not be ignored, as triglycerides of around 10 mmol/L or higher are associated with a high risk of acute pancreatitis.

Before referring a patient, secondary causes such as lifestyle, hypothyroidism, glucose intolerance, liver and renal disease should be excluded and addressed if appropriate.

## Future agents

Clinical trials are currently underway for agents targeting other pathways and also in drug delivery. Cholesteryl ester transfer protein (CETP) inhibitors act to increase HDL-c. Historically, many compounds failed phase III clinical trials and none have yet received market approval. A third-generation agent, obicetrapib, has shown LDL-c reductions of 29.9–31.9% alone and 48.6% with ezetimibe in the TANDEM and BROADWAY studies, and the PREVAIL study looking at MACE outcomes will complete next year.^36^, ^37^, ^38^ There are new developments in drug delivery with oral PCSK9 inhibitors, and gene editing therapy targeting ANGPTL3 and PCSK9, the latter showing durable effects over 12 months after a single infusion.^39^, ^40^, ^41^

## Conclusion

Lipid optimisation leads to significant MACE reduction. Familiarity with all available treatment options including non-statins and injectables will facilitate best primary and secondary care. Lipid lowering is a public health issue, and concerted action is required for long-term gain.

## CRediT authorship contribution statement

**Jennie Han:** Writing – review & editing, Writing – original draft, Visualization, Conceptualization. **Eva Masmanian:** Writing – review & editing, Visualization. **Timothy Wang:** Writing – review & editing, Visualization. **Tuan Peng Chua:** Writing – review & editing, Visualization, Conceptualization.

## Funding

This research did not receive any specific grant from funding agencies in the public, commercial or not-for-profit sectors.

## Declaration of competing interest

Tuan Peng Chua has received an honorarium for presenting a talk on quantitative flow ratio (QFR), a functional assessment of coronary stenosis based on computation analysis of a standard invasive coronary angiogram. Tim Wang has received honoraria from Novartis and Daiichi Sankyo for presentations. Jennie Han and Eva Masmanian have no declarations of interest.
